# Biochanin A exerts an anti-inflammatory effect on adipose tissue and liver of ovariectomized obese mice

**DOI:** 10.1590/1414-431X2025e14737

**Published:** 2025-11-14

**Authors:** J.M.D.A. Aragão, L. Heimfarth, W.S. Neres, F.B. Felix, P.R. dos Santos, F.F. Abreu, L.M. Cercato, A.C.S. Nascimento, A.B.S. Vasconcelos, R.C. Soares, R.L.C. de Albuquerque, G.I. Heiden, T.R. de Moura, E.A. Camargo, R. Grespan

**Affiliations:** 1 Programa de Pós-Graduação em Ciências Fisiológicas, Departamento de Fisiologia, Universidade Federal de Sergipe, São Cristóvão, SE, Brasil; 2 Departamento de Biofísica, Universidade Federal do Rio de Janeiro, Rio de Janeiro, RJ, Brasil; 3 Departamento de Morfologia, Instituto de Ciências Biológicas, Universidade Federal de Minas Gerais, Belo Horizonte, MG, Brasil; 4 Departamento de Medicina, Universidade Federal de Rondônia, Porto Velho, RO, Brasil; 5 Programa de Pós-Graduação em Ciências da Saúde, Departamento de Medicina, Hospital Universitário, Universidade Federal de Sergipe, Aracajú, SE, Brasil; 6 Departamento de Educação em Saúde, Universidade Federal de Sergipe, Lagarto, SE, Brasil; 7 Departamento de Morfologia, Universidade Federal de Sergipe, São Cristóvão, SE, Brasil; 8 Programa de Pós-Graduação em Odontologia, Centro de Ciências da Saúde, Departamento de Patologia, Universidade Federal de Santa Catarina, Florianópolis, SC, Brasil

**Keywords:** Phytoestrogens, High-fat diet, Inflammation, Female castration, Post-menopause

## Abstract

Biochanin A (BCA), a phytoestrogen with broad therapeutic potential, is a promising molecule for alleviating post-menopausal symptoms and treating disorders related to reproductive metabolism. Nevertheless, the effect of BCA on inflammatory changes caused by postmenopausal obesity is unclear. Thus, this study focused on investigating the impact of BCA on the adipose tissue and liver of ovariectomized (OVX) mice subjected to a high-fat diet (HFD). We found that BCA treatment reduced the crown-like structures (CLS), adipocyte area, and hypertrophic adipocyte distribution. This was accompanied by an increase in the anti-inflammatory cytokines interleukin (IL)-5 and IL-10 and the expression of *Mrc1* (CD206), a marker for M2 macrophages. Furthermore, there was a reduction in the extent of hepatic steatosis, triglyceride content, and the expression of *Nos2*, the M1 marker. We concluded that BCA exerted an anti-inflammatory response in the tissues, promoting a resolving profile, although the metabolic profile of the animals was not altered. This study was the first to demonstrate the anti-inflammatory effect of BCA in ovariectomized animals with established obesity.

## Introduction

Overweight and obesity were responsible for 5 million deaths in 2019 due to associated comorbidities such as cardiovascular diseases, diabetes, cancer, and other disorders (1). Obesity results in a pro-inflammatory state in various tissues, starting in metabolically active cells such as adipocytes and hepatocytes, activating inflammation and immune response signaling pathways (2).

The risk of metabolic alterations associated with obesity increases in post-menopausal women due to the reduction of endogenous estrogen, which has a detrimental effect by increasing inflammation (3) and the risk of cardiovascular, hepatic, and diabetic diseases (4).

Estrogen modulates adipose tissue homeostasis. It increases the sensitivity of adipose cells to insulin and the oxidative capacity of the tissue during adipogenesis (5,6). Furthermore, in the experimental model of ovariectomy-induced menopause, estrogen increases the expression of anti-inflammatory macrophage markers (7).

It is well known that the liver is a vital target for estrogen signaling. Ovariectomized mice with non-diet-induced fatty liver disease show significant impairment in hepatic mitochondrial protection (8). Therefore, the physiological actions of estrogen on energy balance, immunity, and immunometabolism become even more important in post-menopause to promote metabolic homeostasis (9).

Hormone replacement therapy (HRT) with estrogens presents several beneficial effects (10). However, HRT has several contraindications, such as an increased risk of cardiovascular events and breast cancer (11). The “timing hypothesis” was proposed to explain that the increased risk of coronary heart disease associated with HRT is related to the timing of its initiation (12). The late estrogen therapy after arteries have already become senescent may pose a risk to the vasculature by increasing the production of contractile factors and promoting vasoconstriction, as observed in ovariectomized mice (13). Nevertheless, the risk of HRT in women near the onset of menopause is not ruled out. This indicates the narrow therapeutic window and safety margin of HRT use.

Therefore, there is a growing interest in safer molecules such as phytoestrogens to reduce the use of estrogen-based HRT or minimize the adverse effects. These phytoestrogens include biochanin A (BCA), an isoflavone isolated from the leaves and stems of *Trifolium pretense L*., widely used to alleviate postmenopausal problems (14). BCA has shown antioxidant, anti-cancer, anti-inflammatory, anti-diabetic, hepatoprotective, and other biological activities that have been extensively investigated (15).

BCA is a potent molecule with effects through multiple signal transduction pathways involved in cell differentiation, inflammation, and metabolism (16). However, there is no evidence regarding the anti-inflammatory effect of BCA in post-menopausal obesity. Therefore, this study aimed to investigate the BCA effects in ovariectomized obese mice with a specific focus on adipose tissue and the liver.

## Material and Methods

### Chemicals and drugs

Ketamine (100 mg/kg) and xylazine were purchased from Syntec (Brazil), biochanin A (BCA) and dimethyl sulfoxide (DMSO) were purchased from Merck (Germany), and D-glucose was purchased from Neon (Brazil).

### Animals

Twenty-eight 17-20 week old healthy, non-pregnant female C57BL/6 mice were obtained from the Central Animal House of the Federal University of Sergipe (UFS). The animals were kept in polypropylene cages (29×18×16 cm) in groups of 2 to 3 per cage in a room with controlled temperature (23±2°C), air exhaust system, and 12-h light/dark cycle, with food and water *ad libitum*. The animals were divided in groups based on the average weight of each cage. The experimental protocols were approved by the Animal Research Ethics Committee of the Federal University of Sergipe under registration numbers 57/16 and 14/18.

### Ovariectomy

The mice underwent bilateral ovariectomy (OVX) surgery (n=21). After trichotomy and asepsis of the region, the ovaries were removed through a dorsal incision with the animals under anesthesia with ketamine (100 mg/kg) and xylazine (10 mg/kg). Seven mice underwent sham surgery, in which the ovaries were exposed and immediately replaced. After that, the animals were kept in cages for 15 days before starting the induction of obesity.

### Obesity induction and treatment

**Figure 1:**
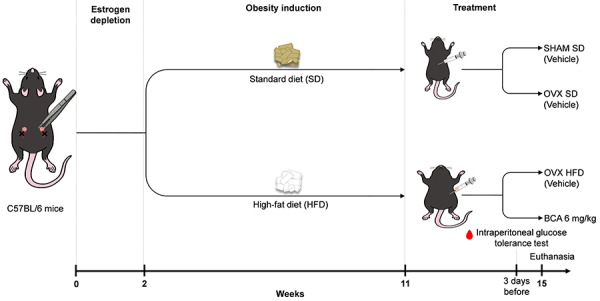
Experimental design. The animals were ovariectomized (OVX) or sham-operated (SHAM) and subjected to their respective diets. After obesity induction, they were treated either with vehicle (saline and 0.5% DMSO) or biochanin A (BCA).

The high-fat diet (HFD) consisted of 26% carbohydrate, 59% lipids, and 15% protein, with lard as the lipid (PragSoluções Biociências^©^, Brazil). The standard diet (SD) consisted of 62% carbohydrates, 13% lipids, and 25% proteins. Both diets contained a mix of vitamins and minerals according to AIN-93 recommendations. The SHAM and OVX SD groups received the SD, while the OVX HFD and BCA groups received the HFD. The experimental design is shown in Figure 1. BCA (6 mg/kg *ip*) was diluted in saline and 0.5% DMSO. The other groups received 100 µL vehicle *ip* (saline and 0.5% DMSO) during the same period. Body weight was measured twice a week to calculate the average for each animal. The animals were euthanized with an overdose of ketamine (100 mg/kg) and xylazine (10 mg/kg), after which blood, adipose tissues, liver, and uterus were collected. To confirm obesity, we calculated the Lee index, which is defined as the cube root of body weight (g) divided by the nasal-anal length (cm), multiplied by 1000 (17).

### Intraperitoneal glucose tolerance test

Mice were fasted for 5 h for the glucose tolerance test. Three days before euthanasia, 1.5 mg/g of animal weight of D-glucose was injected intraperitoneally, and glucose was measured in a drop of blood from the tail vein at 0, 15, 30, 60, and 120 min post-injection with the aid of a glucometer (Bioland G500, Controller-SC¯, Brazil).

### Biochemical parameters

Blood was collected from the retro-orbital plexus after a 10-h fast. The blood was centrifuged at 10,061 *g* at 10°C for 10 min to separate the serum, which was then stored at -20°C for later analysis. The concentrations of glucose and total cholesterol were investigated using commercial kits, following the manufacturer's instructions (Labtest¯, Brazil).

### Histology

Tissues were weighed and samples of perigonadal adipose tissue (AT) were collected from the same anatomical region in all animals and stored in 4% paraformaldehyde for 6 to 24 h at 5°C. Liver samples were kept in 10% buffered formalin at 25°C for at least 24 h for fixation. Tissues were immersed in graded alcohol and xylene and embedded in paraffin. Sections of approximately 5 μm were stained with hematoxylin-eosin (HE), observed with an optical microscope, and analyzed by a blinded evaluator.

### Adipose tissue analysis

The images of the perigonadal adipose tissue were used to count crown-like structures (CLS) and adipocyte areas. Using ImageJ software (NIH, USA), adipocytes were assessed by drawing ellipses around the cell membranes. The software scale was set at 100-μm at 400× magnification. In each analyzed image, 5 to 10 adipocytes were circumscribed, totaling at least 100 adipocytes per mouse. A covert evaluator counted the CLSs along the entire length of the tissue and the result was normalized to 50 fields.

### Liver analysis

To determine the percentage of hepatic steatosis in liver samples, four histological sections of each specimen were analyzed. In each section, ten histological fields of 0.06 mm^2^ (400× magnification) were selected and photographed (Leica DFC340FX digital camera (Germany), coupled to the Olympus CX 31 optical microscope (Japan)). The fields were randomly selected (for each selected field, two fields were neglected). The percentage of liver tissue section area undergoing steatotic vacuolization was calculated using ImageJ. The program was previously calibrated and, once the measurement scale (%) was defined, the measurements were obtained through manual thresholding in grayscale. The total area under analysis (0.06 mm^2^) was considered 100% and the percentage of area identified by the thresholding procedure was subtracted from it to obtain the percentage of steatosis in each histological field (400×). All morphometric measurements were carried out by a blinded evaluator.

### Triglyceride content in the liver

The analysis of total liver fat was carried out according to Folch et al. (18), with a slight modification. Briefly, 1 g of liver tissue was homogenized with 1.5 mL of chloroform: methanol (2:1). The mixture was then filtered through fine filter paper and for every 1 mL of filtrate, 200 μL of saline solution was added. The tubes were gently shaken by inversion three times. Then, the homogenate was centrifuged for 10 min at 956 *g* at room temperature to separate the phases. A known volume of the lower phase (chloroform phase) was transferred to a previously weighed container, placed in the oven (60°C) for evaporation, and resuspended in 1 mL of isopropanol. An aliquot was used for the analysis of total triglycerides (Labtest, Brazil). Finally, the result was normalized by the weight of the tissue used in the analysis.

### Enzyme-linked immunosorbent assay (ELISA)

The pro-inflammatory cytokines interleukin (IL)-6 (Invitrogen™, Brazil) and anti-inflammatory IL-10 and IL-5 (R&D Systems^©^, USA) were quantified in the perigonadal adipose tissue (PGAT), liver, and muscle using ELISA kits, according to the manufacturer's protocol. After that, the result was normalized to the amount of total protein.

### Quantitative real-time PCR

Total RNA was extracted from tissues using Trizol (Thermo Fisher Scientific, USA). RNA quality and concentrations were measured by a spectrophotometer, using the ratio 260/280 nm. RNA (1 µg) samples were reverse transcribed to cDNA using the TaqMan^TM^ High-Capacity cDNA Reverse Transcription Kit (Thermo Fisher Scientific) following the manufacturer's instructions. Relative quantification by real-time PCR was performed using TaqManTM Fast Advanced Master Mix (Thermo Fisher Scientific), in the QuantiStudio^TM^ 5 Real-Time PCR System thermocycler (Thermo Fisher Scientific), under the following conditions: 2 min at 50 °C, 10 min at 95°C, 40 cycles of 15 s at 95°C, and 1 min at 60°C. Amplification data were analyzed using QuantiStudio^TM^ software v1.5.2. TaqMan assays were used to evaluate the genes *Mrc1*, *Arg1,* and *Nos2*, and *Gapdh* (Thermo Fisher Scientific) was used as a housekeeping gene (primers are described in Table 1). Relative quantities were determined using the 2^-ΔΔCt^ method.

**Table 1:** Sequence of primers used in the study.

Primer	Species	Probe context sequence	ID assay
*Mrc1*	*Mus musculus*	ACAGCAACTTGACCAATAATGAATA	Mm01329359_m1
*Nos2*	*Mus musculus*	GCCTTGTGTCAGCCCTCAGAGTACA	Mm00440502_m1
*Arg1*	*Mus musculus*	TTTCTCAAAAGGACAGCCTCGAGGA	Mm00475988_m1
*Gapdh*	*Mus musculus*	GGTGTGAACGGATTTGGCCGTATTG	Mm99999915_g1

### Statistical analysis

Results are reported as means±SEM or median and interquartile range for scores of histological parameters. Statistical analysis of the data was performed using the GraphPad Prism 8 program. The Shapiro-Wilk normality test was used to assess whether the data followed a normal and parametric distribution. One-way ANOVA was performed for independent variables and two-way ANOVA was used for repeated measures. After confirming the normality of the data, Bonferroni's *post hoc* test or Kruskal-Wallis test was applied for multiple comparisons, and P-values <0.05 were considered statistically significant.

## Results

### Effect of BCA treatment on weight and adiposity

First, we confirmed the effectiveness of the ovariectomy based on the uterine index. The SHAM SD animals had a higher uterine index compared to OVX SD, OVX HFD, or BCA (0.39±0.06 *vs* 0.12±0.02, 0.12±0.01, 0.09±0.006; P<0.0001).

**Figure 2:**
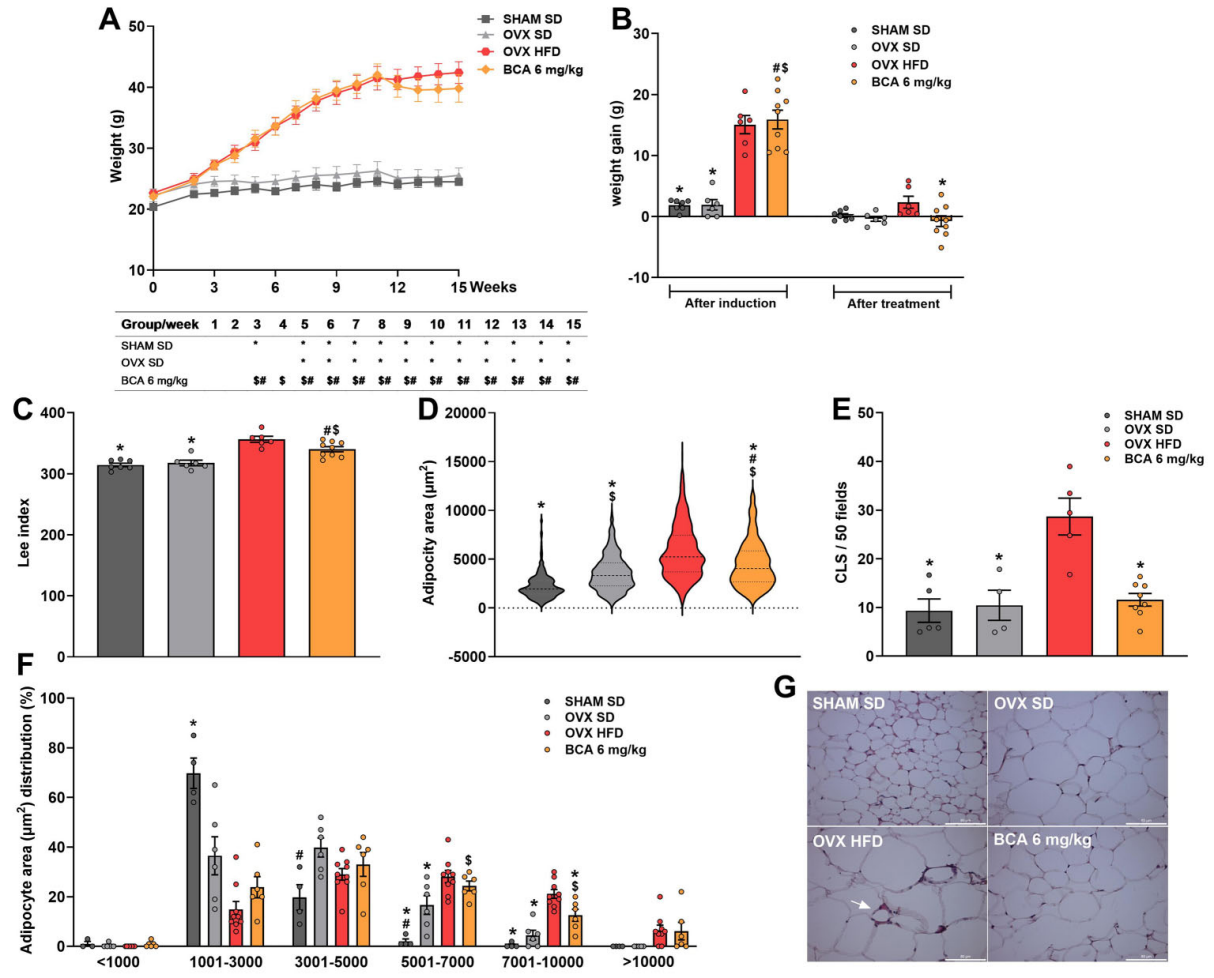
Weight gain and adipose tissue characteristics of the animals. **A**, Evolution of body weight over the course of 15 weeks. **B**, Cumulative weight gain after obesity induction and treatment. **C**, Adiposity index. Characteristics of perigonadal adipose tissue regarding (**D**) area of 100 adipocytes, (**E**) count of crown-like structures (CLS), and (**F**) distribution of adipocyte areas. **G**, Representative images of each group showing CLS (white arrow) (scale bar=80 µm). Data are reported as means±SEM. ^$^P<0.05 *vs* SHAM SD; ^#^P<0.05 *vs* OVX SD; *P<0.05 *vs* OVX HFD. One-way ANOVA followed by Bonferroni (**A**) or two-way ANOVA, followed by Kruskal-Wallis post-test (**B**-**F**). OVX: ovariectomized; SHAM: sham-operated; SD: standard diet; HFD: high-fat diet; BCA: biochanin A (6 mg/kg).

The weight of the animals that received HFD (OVX HFD and BCA) increased from the third week compared to the SD groups (SHAM SD and OVX SD). However, the weight of the animals in the SHAM SD and OVX SD groups did not differ (Figure 2A). Figure 2B shows the weight gain after obesity induction and treatment (at the end of 15 weeks). The SHAM SD and OVX SD groups had lower weight gain compared to the HFD groups (P<0.001 *vs* SHAM SD or OVX SD). After treatment with BCA, the animals showed lower weight gain compared to the OVX HFD group (P=0.036) (Figure 2B).

The OVX HFD group had a higher Lee index compared to the SD groups (P<0.0001, P>0.0001), and BCA treatment did not reduce this index compared to the OVX HFD group (P=0.061) (Figure 2C).

Despite not finding a reduction in weight or adiposity after treatment with BCA, the area and distribution of adipocytes showed interesting results (Figure 2D, F, and G). As expected, the OVX HFD group showed a larger adipocyte area compared to the SD groups (P<0.0001, P<0.0001). Interestingly, the adipocyte area of the OVX SD animals was also larger compared to the SHAM SD group (P<0.0001). Treatment with BCA reduced the adipocyte area compared to the OVX HFD group (P<0.0001), but it was still larger than the SD groups (P<0.0001 and P<0.0001) (Figure 2D).

Additionally, we evaluated the distribution of adipocytes in the PGAT. The SHAM SD group had a higher percentage of adipocytes with an area of 1001-3000 µm^2^ *vs* the OVX HFD group (P=0.0018). As the area of adipocytes increased to 5001-7000 µm^2^, the percentage of adipocytes remained higher for the OVX SD compared to SHAM SD animals (P=0.02). The OVX HFD group had a higher percentage of adipocyte distribution compared to SHAM SD and OVX SD groups (P<0.0001, P=0.0281). The group treated with BCA had a higher percentage of distribution than the SHAM SD (P=0.0003). The adipocytes with areas of 7001-10,000 µm^2^ were more prevalent in OVX HFD than in SHAM SD, OVX SD, and BCA groups (P<0.0001, P<0.0001, P=0.022), but the BCA group still had a higher percentage than the SHAM SD group (P=0.0076). With BCA treatment, we found a reduction in weight gain, adipocyte area, and number of hypertrophied (7001-10,000 µm^2^) adipocytes.

To assess the relationship between propensity for tissue inflammation and adipocyte size, the quantity of CLSs in the PGAT was evaluated. The SD groups had fewer CLSs in adipose tissue than the OVX HFD group (P=0.0003). Additionally, BCA treatment reduced CLSs to normal levels (BCA *vs* SHAM SD, P=0.99; BCA *vs* OVX HFD group, P=0.0004) (Figure 2E).

### Effect of BCA on hyperglycemia and hypercholesterolemia

**Figure 3:**
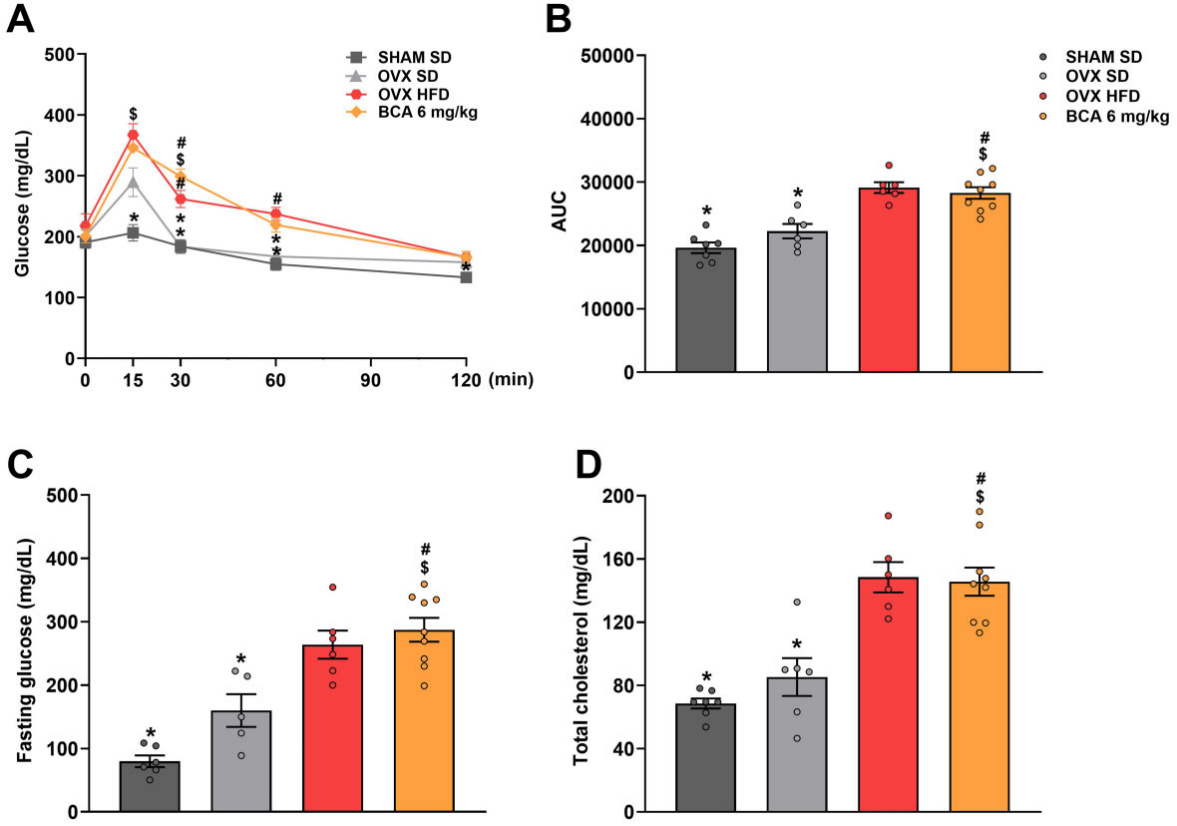
Assessment of the metabolic profile of the animals. **A**, Glucose tolerance test was performed after a 5-h fasting. Blood glucose was measured at 0, 15, 30, 60, and 120 min after the administration of D-glucose (1.5 mg/g of weight). **B**, Area under the test curve (AUC). **C**, Fasting glucose and (**D**) total cholesterol were also measured after a 10-h fast. Data are reported as means±SEM. One-way ANOVA, followed by Bonferroni's post-test (**A**) or two-way ANOVA, followed by Bonferroni's post-test (**B**-**D**). ^$^P<0.05 *vs* SHAM SD; ^#^P<0.05 *vs* OVX SD; *P<0.05 *vs* OVX HFD. OVX: ovariectomized; SHAM: sham-operated; SD: standard diet; HFD: high-fat diet; BCA: biochanin A.

Although blood glucose was similar among the groups, the OVX HFD group showed increased glucose levels compared with the SHAM SD group, starting at 15 min and persisting until the end of the test (P=0.003, P=0.0081, P=0.0011, P=0.0027). BCA treatment also resulted in elevated blood glucose at all time points (P<0.0001, P<0.0001, P=0.0057), except during the final 120 min (P=0.0691). The OVX SD animals exhibited lower blood glucose levels compared to the OVX HFD group (30 and 60 min) (P=0.010, P=0.018) (Figure 3A).

Corroborating these results, the area under the curve (AUC) was higher for the OVX HFD group than the SD groups (P<0.0001, P=0.0005). BCA treatment did not show a significant difference with OVX HFD (Figure 3B).

Fasting blood glucose and total cholesterol showed a similar pattern of results among the groups. Compared to the SD groups, glucose, and cholesterol were higher in the OVX HFD (P<0.0001, P=0.0154) and BCA group (P<0.0001, P=0.0006) (Figure 3C and D).

### BCA treatment improves steatosis in the liver

**Figure 4:**
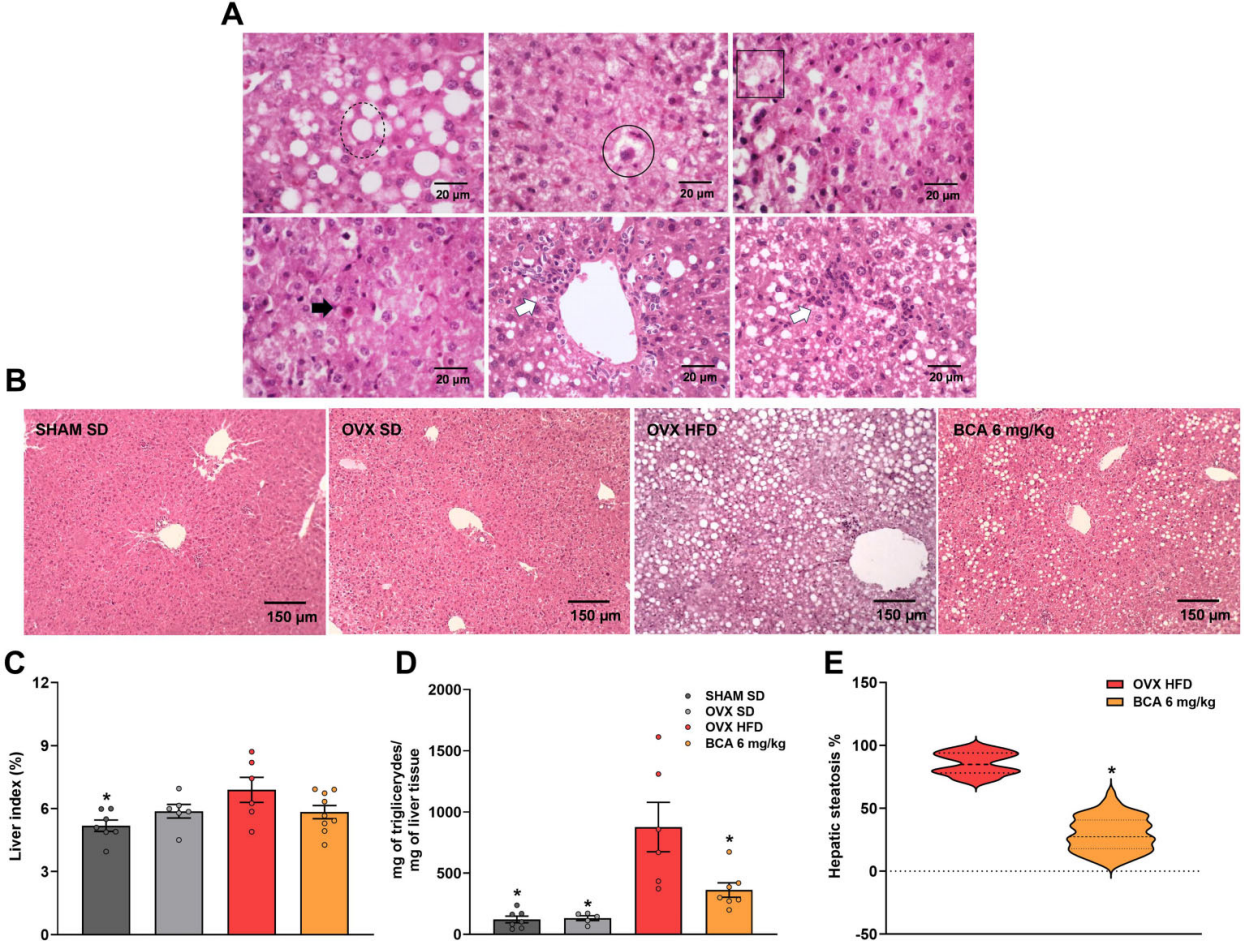
Influence of BCA treatment on histopathological changes in the liver. Livers were stained with HE and the images were taken at 400× magnification. **A**, Fat vacuoles (dotted circle), microvesicular steatosis (continuous circle), cell ballooning (square), apoptotic body (black arrow), and mononuclear cells (white arrows) are shown in the centrilobular (bottom second) and intralobular (bottom third) veins (scale bars=20 µm). **B**, Representative images of each groups (scale bar=150 µm). **C**, Liver index. **D**, Triglycerides concentration normalized by total tissue weight (mg). **E**, Percentage of liver steatosis in the groups that received the HFD. Data are reported as means±SEM or as violin plots with median titers and the range of 5-95%. One-way ANOVA followed by the Bonferroni's post-test (**C** and **D**) or Kruskal-Wallis post-test (**E**). *P<0.05 *vs* OVX HFD. OVX: ovariectomized; SHAM: sham-operated; SD: standard diet; HFD: high-fat diet; BCA: biochanin A.

Histopathological evaluation of liver tissue showed an accumulation of large intracytoplasmic fat vacuoles (macrovesicular steatosis), promoting cell ballooning. Areas of microvesicular steatosis, characterized by multiple intracytoplasmic lipid vacuoles and apoptotic bodies, were evident to a lesser extent. Mononuclear inflammatory infiltrate was observed in all groups that consumed HFD, but it was considered mild and had an irregular distribution (Figure 4A). Despite these findings, the liver index was different only between the SHAM SD and OVX HFD groups, with the latter showing an increase of about 36% (P=0.02) (Figure 4C).

As expected, the OVX HFD group had a higher content of triglycerides in the liver than the SD groups (P=0.0002, P=0.0006, P=0.009). The treatment with BCA reduced triglycerides compared to OVX HFD group (P=0.009) to a concentration similar to that in the SD groups.

**Figure 5:**
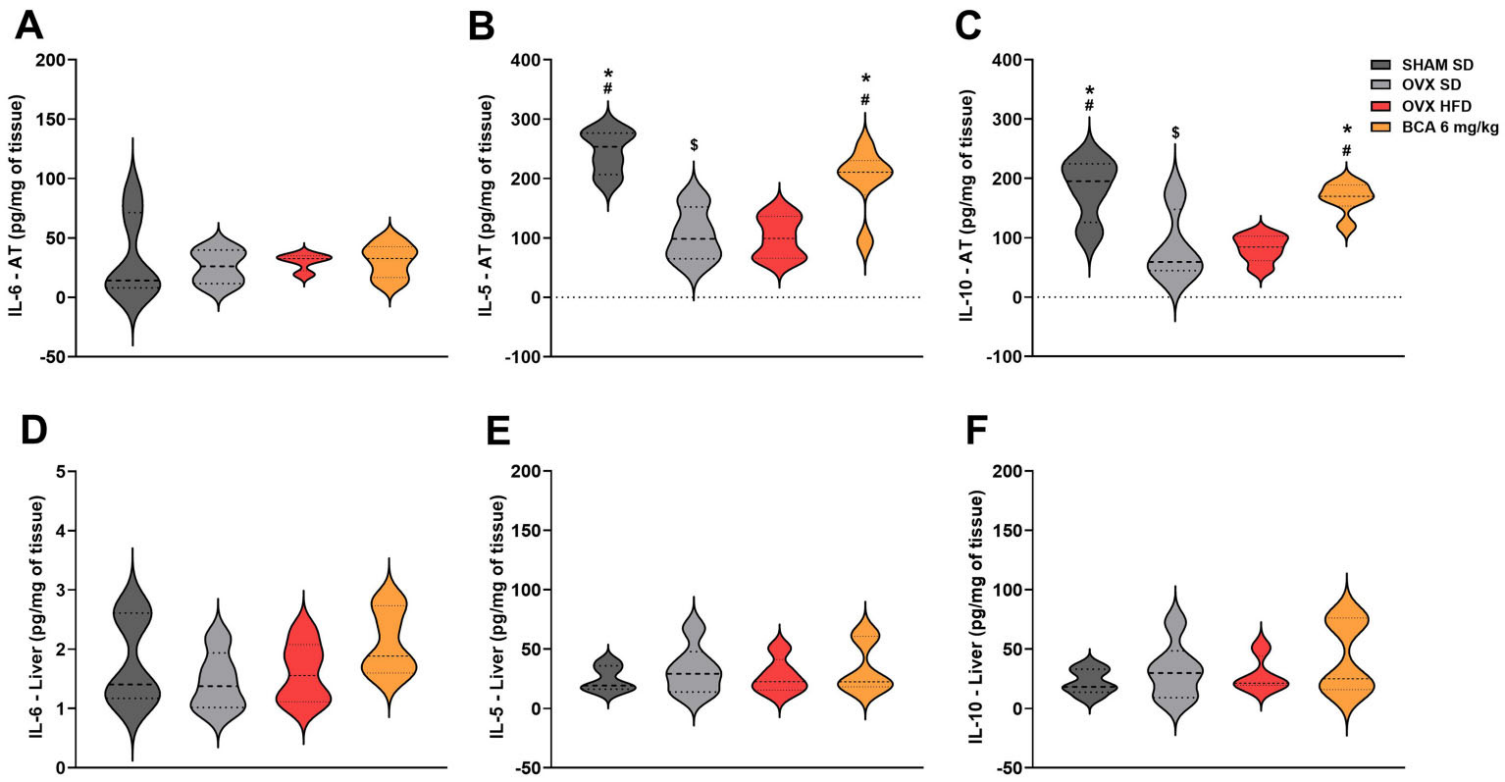
Profile of cytokines in adipose tissue (AT) and liver. Pro-inflammatory cytokine interleukin (IL)-6 and anti-inflammatory cytokines IL-5 and IL-10 were evaluated in perigonadal adipose tissue (**A**-**C**) and liver (**D**-**F**). Data are reported in violin plots with median titers and 5-95% range. One-way ANOVA followed by Bonferroni or Kruskal-Wallis post-test was used. ^$^P<0.05 *vs* SHAM SD; ^#^P<0.05 *vs* OVX SD; *P<0.05 *vs* OVX HFD. OVX: ovariectomized; SHAM: sham-operated; SD: standard diet; HFD: high-fat diet; BCA: biochanin A.

BCA treatment reduced hepatic steatosis compared with the OVX HFD group (P<0.0001) (Figure 4E). The representative images clearly show this effect (Figure 4B). Interestingly, the concentration of cytokines did not change in the liver (Figure 5D-F).

### Effect of BCA on adipose tissue and liver inflammation

**Figure 6:**
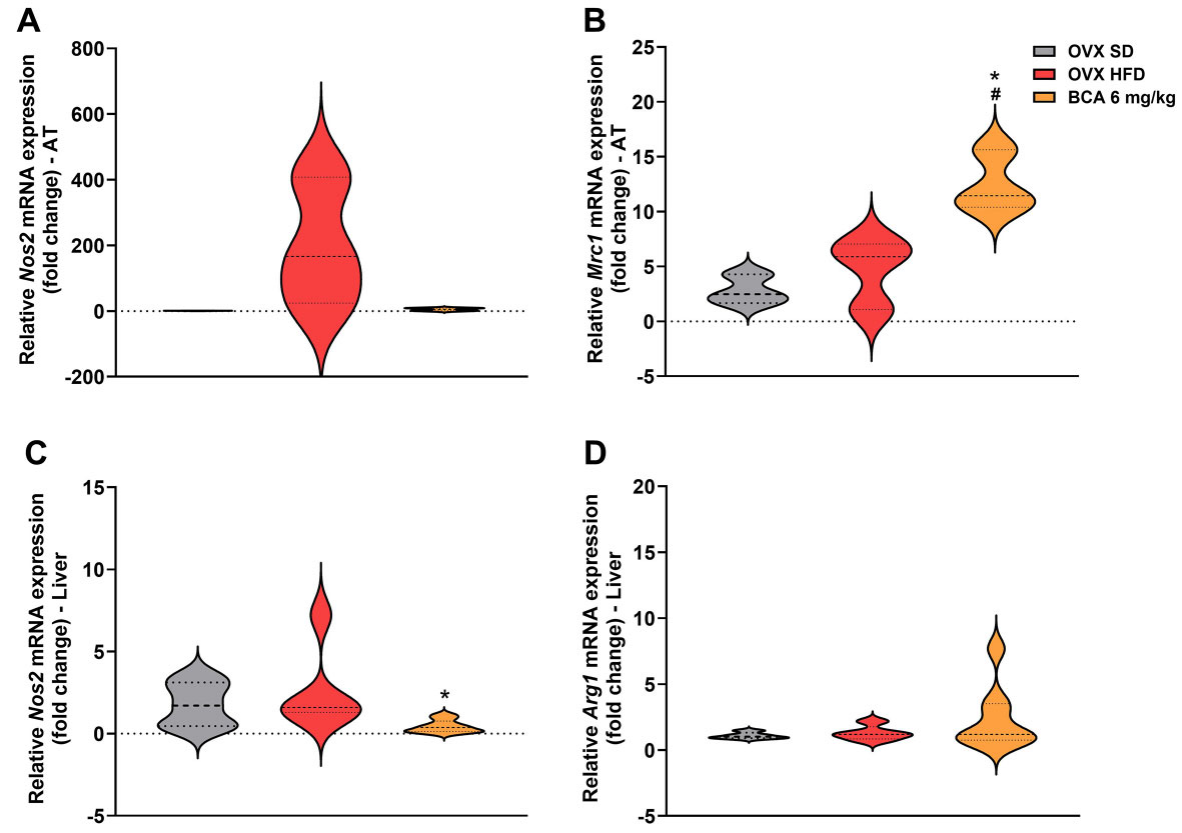
Expression of genes linked to inflammation and macrophage profile in tissues. Gene expression of *Nos2* in adipose tissue (AT) (**A**) and liver (**C**), related to the M1 macrophage profile, and *Mrc1* (**B**) or *Arg1* (**D**), as genes linked to the M2 profile. Data are reported as means±SEM. ^#^P<0.05 *vs* OVX SD; *P<0.05 *vs* OVX HFD. One-way ANOVA followed by Bonferroni's post-test. OVX: ovariectomized; SHAM: sham-operated; SD: standard diet; HFD: high-fat diet; BCA: biochanin A.

The results of the inflammation-related cytokines are shown in Figure 5. The anti-inflammatory cytokines IL-5 and IL-10 (Figure 5B and C) were reduced in the OVX HFD *vs* SHAM SD group (P=0.0015, P=0.0079). Additionally, the OVX SD group also showed a reduction in these cytokines compared to the SHAM SD group (P=0.0031, P=0.0191). BCA treatment reversed the depletion of these cytokines compared to the OVX SD group (P=0.0359, P=0.0312) and the OVX HFD group (P=0.0169, P=0.0113). Reinforcing the anti-inflammatory effect of BCA, these results were associated with an increase in the expression of anti-inflammatory markers such as *Mrc1* (BCA *vs* OVX SD and OVX HFD; P=0.0105, P=0.0282; Figure 6B).

IL-6 concentration (Figure 5A) in adipose tissue did not change among the groups. Corroborating this result, there were no differences in *Nos2* expression (Figure 6A).

Finally, the anti-inflammatory effect of BCA in the liver was also observed, indicated by a reduced expression of *Nos2* in the BCA group compared with the OVX HFD group (P=0.01) (Figure 6D).

## Discussion

The present study investigated the potential anti-inflammatory effects of BCA treatment in a murine model of obesity induced by ovariectomy. In summary, our findings indicated that BCA treatment reduced inflammation in adipose tissue and decreased adiposity area, despite not affecting overall body weight or the Lee index. BCA treatment also increased the concentrations of the anti-inflammatory cytokines IL-5 and IL-10 in adipose tissue, likely associated with a reduction in the expression of biomarkers for M1 macrophages (pro-inflammatory phenotype). We hypothesize that this anti-inflammatory effect on adipose tissue may be linked to macrophage polarization.

In the liver, BCA treatment decreased steatosis and triglyceride content in the high-fat diet-induced obesity mice and reduced molecular biomarkers for M1 macrophages, even though it did not significantly modulate inflammatory cytokine levels. BCA has previously been investigated in cell lines, mice, and male rats with obesity induced by diet, with claims of anti-obesity and anti-inflammatory effects (19-21). To the best of our knowledge, this study is the first to evaluate the anti-inflammatory effects of BCA in a model of established obesity in OVX animals.

Ovariectomy is a widely used surgery in animal studies on depletion of endogenous estrogen (oophorectomy in humans), mimicking the physiological condition of menopause. The hypoestrogenic effect of ovariectomy leads to increased visceral fat, inflammation, oxidative stress, and metabolic complications such as dyslipidemia, hepatic steatosis, and predisposition to cardiovascular diseases (3,22). Our study showed an increase in weight and adiposity and worsened glycemic and lipid profiles of the OVX HFD animals.

Oza and Kulkarni (23) showed BCA's anti-hyperglycemic effect in obese and diabetic rats. Although BCA did not modulate lipid and glucose metabolism, a closer examination of its effects revealed that BCA reduced weight gain, adipocyte area, and the number of hypertrophied adipocytes. Thus, in a subtle and likely preliminary manner, we demonstrated the anti-obesity activity of BCA. Additionally, we observed a significant anti-inflammatory effect of BCA in this tissue.

During obesity development, the expansion of adipose tissue culminates in a network of inflammatory and metabolic events that challenge proposed therapies. Soon after HFD consumption and before adipocyte hypertrophy, there is an increase in neutrophils that induce insulin resistance (IR) (24). After weeks of HFD consumption, the flow of saturated fatty acids into adipose tissue leads to inflammation, monocyte recruitment, and polarization to M1 macrophages (25,26). These highly inflammatory macrophages form CLSs and release chemokines and cytokines that recruit more leukocytes (27). CLSs are a pathological hallmark of obesity, indicating that adipocyte residue elimination is a key function of macrophages in obese individuals. Adipocyte death is positively correlated with increased adipocyte size in obese mice (28).

Adipose tissue is regulated by immune molecules and cells that determine the inflammatory profile, such as eosinophils, which are important producers of Th2 cytokines like IL-5 and IL-10, supporting metabolic homeostasis in healthy states (29,30). Lee et al. (30) observed in eosinophil-deficient mice that these cells are essential for improving tissue metabolic profiles, and they were inversely associated with adiposity and IR (31). Another group reported that restoring these cells to physiological levels induced by IL-5 did not restore glucose tolerance in obese animals (32). The findings of this study support this, as the increase in IL-5 in obese ovariectomized animals treated with BCA did not improve glucose tolerance.

Another significant aspect of inflammation is the profile of macrophages present in the tissue. In this regard, the role of IL-10 in inducing the M2 macrophage profile is well established. In healthy conditions, adipose tissue produces IL-13, promoting the activation of M2 macrophages and suppressing M1 macrophages by producing adiponectin and IL-10 (33). This cytokine, secreted by regulatory T lymphocytes, has anti-inflammatory effects, increasing insulin sensitivity and opposing the role of tumor necrosis factor (TNF)-α in inducing insulin resistance in 3T3L adipocytes (25).

A likely shift toward the M2 macrophage profile was observed in the adipose tissue of BCA-treated animals, represented by the increase in anti-inflammatory cytokines IL-5 and IL-10 and the gene expression of CD206 (*Mrc1* gene), linked to the M2 macrophage phenotype (34). CD206^+^ M2 macrophages in adipose tissue have been associated with a microenvironment that inhibits the growth and differentiation of progenitor adipocytes, controlling adiposity and systemic insulin sensitivity (35).

In our study, we did not observe changes in the concentration of the pro-inflammatory cytokine IL-6, which made it challenging to detect inflammation in OVX animals. However, as previously reported, CLS is a hallmark of inflammation in adipose tissue. Additionally, Williams et al. (36) investigated the stages of obesity development in mice induced by a high-fat diet and found no changes in IL-6 expression in adipose tissue or plasma cytokine levels after 16 weeks of diet consumption. These findings were associated with an increase in the expression of anti-inflammatory markers such as *Mrc1* (CD206) and IL-10, similar to what we observed in our study.

The anti-steatosis effect of BCA was previously investigated by Park et al. (37), showing that the phytoestrogen promoted the recovery of metabolites linked to lipogenesis and beta-oxidation and suppressed the expression of enzymes involved in glucose metabolism in male mice.

Regarding the unchanged cytokine concentration in the liver, Williams et al. (36) also did not observe alterations in the expression of pro-inflammatory markers in this tissue at any of the time points evaluated, suggesting an early inflammatory response. The kinetics of chronic inflammatory response in the liver was investigated by Bae et al. (38), who found that inflammation-related genes are highly expressed after 2 weeks of diet, peak at 8 weeks, and then abruptly decline between 16 and 20 weeks. Thus, this may account for the lack of effect of BCA on hepatic inflammation.

BCA negatively modulated the expression of the *Nos2* gene in pro-inflammatory macrophages in the animals' livers. This gene encodes inducible nitric oxide synthase (iNOS), an enzyme expressed in Kupffer cells in non-alcoholic fatty liver disease and is strongly associated with insulin resistance due to impaired autophagy in hepatocytes during diet-induced obesity (39). Although more data are needed, these findings suggest that the hepatic protection provided by BCA against steatosis may be linked to the reduced expression of this enzyme.

The anti-inflammatory effect of BCA is believed to work by modulating various targets to prevent/reduce the inflammatory process, such as the inhibition of NF-κB and MAPK through the upregulation of PPAR-γ (15,16). Additionally, investigation into the modulation of STAT3 to regulate macrophage polarization via the JAK/STAT pathway deserves special attention, as it is an extremely important pathway for this effect (40). Our study did not cover these investigations and therefore we emphasize the need for future studies to address this gap and investigate the specific signaling pathways responsible for BCA's effects on macrophage polarization in adipose tissue and liver exposed to obesity.

## Conclusion

Our results showed that the effect of BCA was not directly linked to its anti-hyperlipidemic and anti-hyperglycemic effects, as seen in previous studies, but to its anti-inflammatory properties, improving the macrophage profile in adipose tissue and the liver. Therefore, the mechanisms behind these effects, possibly related to inflammation resolution, need further investigation. The study has some limitations, including the route of administration and dose used, but it presents important findings and contributions that indicate the need for further investigation into the effects of BCA on obesity during postmenopause.

## Data Availability

All data generated or analyzed during this study are included in this published article.
